# Supplementary material for: Novel Driver Strength Index highlights important cancer genes in TCGA PanCanAtlas patients
Source: PeerJ. 2022 Aug 11;10:e13860. doi: 10.7717/peerj.13860 (PMC9375969; doi:10.7717/peerj.13860)
Supplement: Supplemental Information 2 [file peerj-10-13860-s002.zip › COHORTS/genes plots/detailed/2021_8_16_14_9_distribution_events_detailed_males_11.pdf]

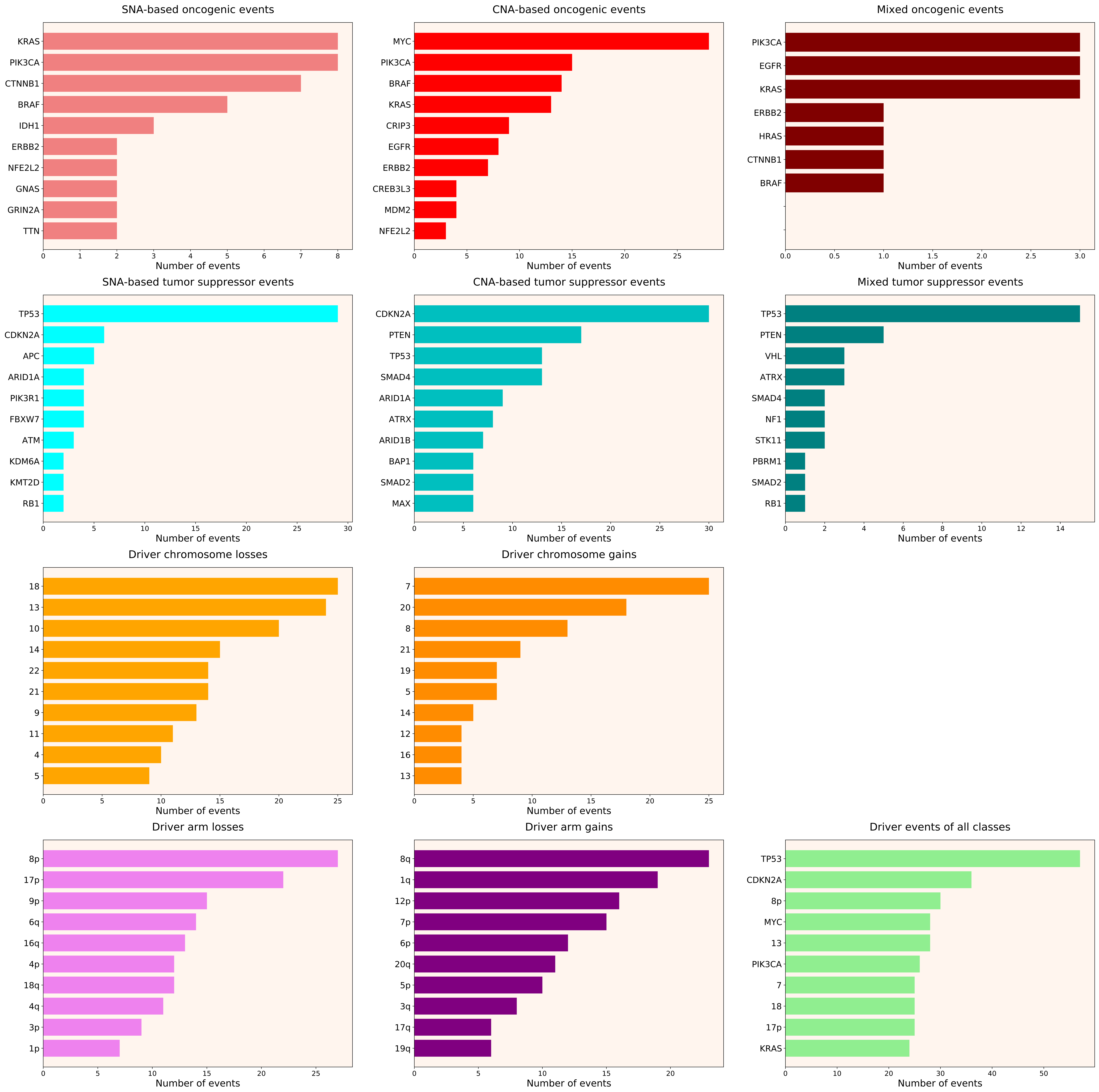
SNA-based oncogenic events
KRAS
PIK3CA
CTNNB1
BRAF
IDH1
ERBB2
NFE2L2
GNAS
GRIN2A
TTN
Number of events
CNA-based oncogenic events
MYC
PIK3CA
BRAF
KRAS
CRIP3
EGFR
ERBB2
CREB3L3
MDM2
NFE2L2
Number of events
Mixed oncogenic events
PIK3CA
EGFR
KRAS
ERBB2
HRAS
CTNNB1
BRAF
Number of events
SNA-based tumor suppressor events
TP53
CDKN2A
APC
ARID1A
PIK3R1
FBXW7
ATM
KDM6A
KMT2D
RB1
Number of events
CNA-based tumor suppressor events
CDKN2A
PTEN
TP53
SMAD4
ARID1A
ATRX
ARID1B
BAP1
SMAD2
MAX
Number of events
Mixed tumor suppressor events
TP53
PTEN
VHL
ATRX
SMAD4
NF1
STK11
PBRM1
SMAD2
RB1
Number of events
Driver chromosome losses
18
13
10
14
22
21
9
11
4
5
Number of events
Driver chromosome gains
7
20
8
21
19
5
14
12
16
13
Number of events
Driver arm losses
8p
17p
9p
6q
16q
4p
18q
4q
3p
1p
Number of events
Driver arm gains
8q
1q
12p
7p
6p
20q
5p
3q
17q
19q
Number of events
Driver events of all classes
TP53
CDKN2A
8p
MYC
13
PIK3CA
7
18
17p
KRAS
Number of events
